# A Systematic Framework for Olfactory Bulb Signal Transformations

**DOI:** 10.3389/fncom.2020.579143

**Published:** 2020-09-23

**Authors:** Thomas A. Cleland, Ayon Borthakur

**Affiliations:** ^1^Computational Physiology Laboratory, Department of Psychology, Cornell University, Ithaca, NY, United States; ^2^Computational Physiology Laboratory, Field of Computational Biology, Cornell University, Ithaca, NY, United States

**Keywords:** spike synchronization, category learning, perceptual learning, olfaction, plasticity, neuromorphic, computational modeling, learning in the wild

## Abstract

We describe an integrated theory of olfactory systems operation that incorporates experimental findings across scales, stages, and methods of analysis into a common framework. In particular, we consider the multiple stages of olfactory signal processing as a collective system, in which each stage samples selectively from its antecedents. We propose that, following the signal conditioning operations of the nasal epithelium and glomerular-layer circuitry, the plastic external plexiform layer of the olfactory bulb effects a process of category learning—the basis for extracting meaningful, quasi-discrete *odor* representations from the metric space of undifferentiated olfactory quality. Moreover, this early categorization process also resolves the foundational problem of how odors of interest can be recognized in the presence of strong competitive interference from simultaneously encountered background odorants. This problem is fundamentally constraining on early-stage olfactory encoding strategies and must be resolved if these strategies and their underlying mechanisms are to be understood. Multiscale general theories of olfactory systems operation are essential in order to leverage the analytical advantages of engineered approaches together with our expanding capacity to interrogate biological systems.

## Introduction

Theoretical models of neural systems serve as proofs of concept, tests of sufficiency, and quantitative embodiments of working hypotheses (Cleland and Linster, 2005). Well-vetted models impose discipline on hypothesis generation, and become particularly important when the systems under study are too complex to simply intuit. However, such models can reflect only the sources of variance that are adequately appreciated by theoreticians during model design. It is axiomatic in engineering that new designs are likely to require revision once physical prototypes are built, often because neglected minor variables generate significant unforeseen effects. This same principle applies to the reverse engineering of biological systems. Biological systems are physical systems that operate autonomously within insecure and incompletely predictable natural environments; their continued existence implies their adequate management of all relevant variables and successful exploitation of pertinent information. Identifying the properties that enable biological systems to adapt successfully to unmitigated natural variance is a core challenge both for our understanding of biological operations and for the design of autonomous robotic systems.

Here, we describe a set of physically explicit systems models of the early olfactory system that constrain and elucidate the neuronal networks that sample chemosensory data from the environment and present information extracted from these data to the central nervous system. This approach emphasizes an integrated appreciation of systems-level operations *in situ*: i.e., given a limited physical toolset of sensors and network elements, what data can be reliably acquired, what useful information can be plausibly extracted therefrom, and what conditions must be met in order to extract this useful information successfully? To date, this reverse engineering strategy has produced predictive models that have stood the test of time—despite their initial construction on relatively sparse direct evidence, and despite substantial revisions, owing to subsequent experimental studies, to our understanding of the cellular and network elements that directly underlie these computations. In particular, physical, engineered instantiations of biological systems models and the utilization of physical sensor data can present designed networks with the full spectrum of underappreciated difficulties that natural systems encounter routinely, thereby probing their capacities to manage unregulated stimuli encountered under unpredictable circumstances.

## Three Core Problems in Engineering Sensory Systems

In sensory systems, this capacity for managing unpredictable input statistics is particularly important, because the external physical environment does not respect the limitations of biological systems, and the process of data sampling lacks the shared assumptions between sender and receiver that underlie the efficient encoding and transmission of information. Sources of potentially informative variance in the physical world are wildly diverse, and biological sensory systems must adapt to acquire the information that they need using tools with imperfect and idiosyncratic properties. These principles strongly constrain and determine the architecture of sensory systems. As a rough analogy, much of the circuitry in a commercial amplifier also is designed to manage unregulated external variance and compensate for the physical limitations of circuit elements so that the core amplifier elements can function properly and predictably in the real world. Neurons also exhibit limited bandwidth, notoriously non-linear response properties, and relatively narrow response ranges to sensory or synaptic input. It is to be expected that many of the circuit elements and properties of early sensory systems exist for the unlovely but essential processes of signal conditioning.

The core problems addressed by sensory systems can be divided into three phases:

1. Sampling the environmental variance of interest adequately, using available sensors, by any means necessary. In particular, the range of intensities of environmental signals often exceeds the response ranges of biological sensor elements by orders of magnitude. This renders it difficult to even encode afferent information, much less to organize it internally once encoded.
2. Identifying the particular aspects of the sampled variance that are relevant to a given sensory or behavioral task, and segregating this information from the uninteresting or confusing aspects of that variance.
3. Transforming the sensory information into a usable format or formats. This includes the problem of rendering this information relatively portable—i.e., usable by other brain systems and integrable with other modalities. Sometimes this may involve reframing such information into a common, abstract metric space.

### Sampling Strategies

All sensory systems diversify their primary sensors in order to adequately sample the relevant environmental variance. Retinal cone photoreceptor sensitivities are diversified along the axis of wavelength, such that together they are able to detect a wider chromatic range of signals and, once detected, identify and distinguish specific colors (Birch and Wright, 1961). Adaptation to a wide range of environmental light levels is achieved by a set of strategies including iris contraction and the reliance on different photoreceptor populations during light-adapted and dark-adapted vision. Similarly, somatosensory tactile mechanoreceptors are diversified along axes of depth (under the skin) and adaptation timescale, as well as physical location on the body, so that in combination a wide range of contact modes, textures and other constructed tactile stimuli can be distinguished.

Nasal chemoreceptors are notoriously diverse, with mammals exhibiting hundreds of different olfactory receptor types in their noses that selectively respond to specific ranges of molecular ligands (Araneda et al., 2000). However, ligand concentration also strongly affects receptor binding and olfactory sensory neuron (*OSN*) activation (Schaefer et al., 2001; Storace and Cohen, 2017), substantially disrupting the naïve odor quality signal based on combinatorial activation patterns. Accordingly, a great deal of attention has been paid to the broad problem of concentration “invariance” in olfaction. However, there has been relatively little attention paid to the closely related problem imposed by the sharply limited dynamic response ranges of individual neurons. How can different odorant concentrations spanning multiple orders of magnitude even be sampled given the Boltzmann limitations on ligand-receptor binding properties (cooperativity ≥1), which restrict the dynamic input range of primary sensory receptors to roughly 1.5 orders of magnitude concentration? And, if this can be solved, how can this signal diversity then be organized and compressed back into a range that can be physically represented by downstream neural activity?

**Figure 1:**
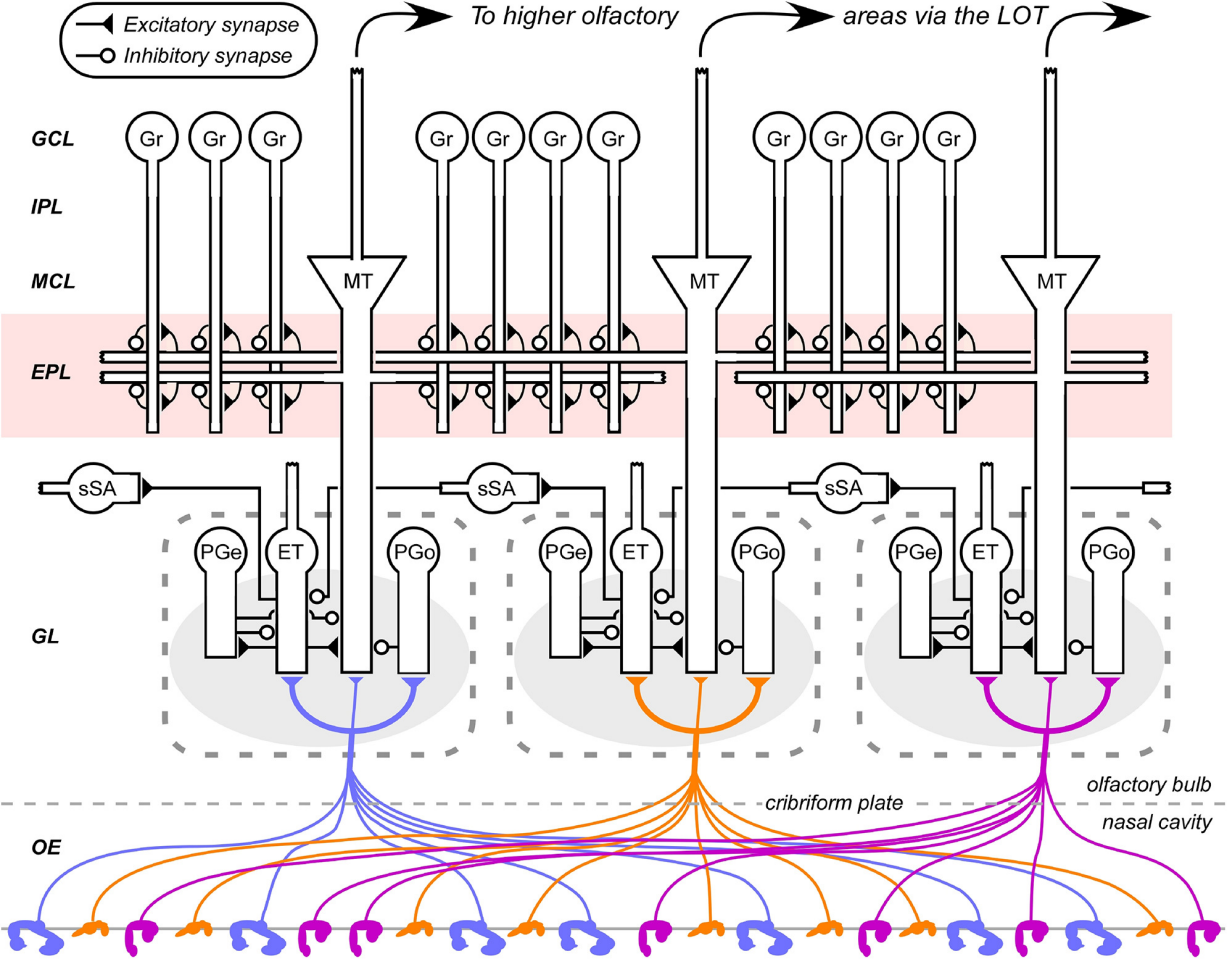
Circuit diagram of the mammalian olfactory bulb. The axons of olfactory sensory neurons expressing the same odorant receptor type converge together as they cross into the brain and arborize together to form glomeruli (shaded ovals) across the surface of the olfactory bulb. Several classes of olfactory bulb neuron innervate each glomerulus. Interneurons include external tufted cells (ET), olfactory nerve-driven periglomerular cells (PGo), and external tufted cell-driven periglomerular cells (PGe). Superficial short-axon cells (sSA) project broadly and laterally within the deep glomerular layer, interacting with glomerular interneurons. Principal neurons include mitral cells and projecting tufted cells (collectively depicted as MT), which interact via reciprocal connections in the external plexiform layer (EPL) with the dendrites of inhibitory granule cells (Gr). Both of these principal neuron types project divergently to several regions of the brain. EPL interneurons and the multiple classes of deep short-axon cell are not depicted. OE, olfactory epithelium (in the nasal cavity); GL, glomerular layer; EPL, external plexiform layer; MCL, mitral cell layer; IPL, internal plexiform layer; GCL, granule cell layer. Filled triangles denote excitatory synapses; open circles denote inhibitory synapses. Note the updates to this diagram compared to its earlier incarnations (e.g., Cleland, 2014).

The answer to this question has not been fully experimentally demonstrated, but theoretical work has demarcated a set of strategies that could underlie this capacity. Briefly, if a large population of sibling OSNs (i.e., those expressing the same odorant receptor) could exhibit diverse EC_50_ values—hence each responding optimally to a different concentration range—then, in combination, their population activity generates a Boltzmann function with a reduced cooperativity and a correspondingly extended dynamic range across concentrations. This diversity can be achieved without loss of specificity if primary OSNs exhibit a range of spare receptor capacities (Cleland and Linster, 1999). The population activity then can be extracted from the glomeruli of the main olfactory bulb (*MOB*), upon which the axons of sibling OSNs converge (Figure 1). Indeed, optical imaging studies have demonstrated that the dynamic ranges of glomerular activation (effectively presynaptic population recordings from convergent OSN axons) extend across many orders of magnitude of odorant concentration, even as the corresponding postsynaptic activity in the apical dendrites of second-order principal neurons (mitral and tufted cells; *MTCs*) remains relatively stable (Storace and Cohen, 2017). These results reveal that the primary OSN population can successfully encode an extensive intensity range of environmental variance, encompassing diverse qualities, and concentrations of some number of odorous ligands. A series of established neurophysiological mechanisms then can compress this wide-range population signal into the limited dynamic range exhibited by activated MTCs (reviewed in Cleland et al., 2012; Cleland, 2014).

### Selecting Relevant Variance

The processes by which primary sensory representations are mined for useful information are hugely diverse and challenging to fully understand. Primary representations generally reflect information derived from multiple conflated causes and sources and are correspondingly difficult to parse. A two-dimensional retinal pattern includes multiple objects of potential interest, occluding one another and perhaps in motion, embedded within regions that may exhibit very different intensities of light and contrast. Odorant receptors are affected by concentration as well as quality; moreover, the ligands that comprise different sources of interest may compete for the same receptors—as agonists, partial agonists, or antagonists—and hence occlude source-specific signal patterns (Gronowitz et al., 2020; Xu et al., 2020; Zak et al., 2020). Indeed, even two source odorants in binary mixtures often strongly interfere with one another, generating activation patterns that do not resemble the diagnostic patterns of any individual source (Linster and Cleland, 2004; Riffell, 2012; Thomas-Danguin et al., 2014). Aside from the specialized mixture interactions literature, most contemporary studies of “odor coding” elide this fundamental problem, focusing instead on single odorants delivered under clean conditions. We argue here that the problem of inter-source interference is fundamentally constraining on early-stage olfactory encoding strategies and must be resolved if these strategies and their underlying mechanisms are to be understood.

We propose that the most effective metaphor for framing the complexities of sensory processing is that neuronal populations comprise computational *stages*, each of which selectively constructs a sensory representation from the activity of its antecedents. That is, after the most peripheral sensory circuits have sampled a wide diversity of information from the environment, secondary sensory circuits sample in turn from this ensemble, deploying specific sampling strategies to selectively extract desired information as if they were themselves primary sensory neurons. Critically, multiple different neuronal populations can extract different information from the same antecedent ensemble, and these divergent pathways then can serve different, if potentially interacting, goals (Milner and Goodale, 2008; Milner, 2017). In the olfactory system, for example, mitral cells, and projecting tufted cells exhibit different response profiles to odors and concentrations (Igarashi et al., 2012; Geramita and Urban, 2017), although both sample from the same primary sensory information presented by MOB glomeruli. Moreover, different subsets of these principal neuron populations project from the olfactory bulb to divergent follower structures—including a lateral pathway to the piriform cortex (PCx) and cortically organized olfactory tubercle, a ventromedial pathway to the striatally organized portion of the olfactory tubercle, and a rostromedial pathway to the tenia tecta and indusium griseum (Ojima et al., 1984; Cleland and Linster, 2019)—and emphasize different features of odor stimuli (Nagayama et al., 2010; Igarashi et al., 2012; Xia et al., 2015). The same metaphor can be applied at a more focused scale as well; for example, one can meaningfully speak of the two computational layers of the olfactory bulb (Cleland, 2014), or even of the properties of the MTC representation vs. those of the granule cell representation as they each evolve along the timecourse of signal processing.

These selective sampling processes are inescapably intertwined with the transformations in the representational metric itself that occur in the MOB. Activity across the population of primary sensory neurons is temporally structured in the theta band (2–8 Hz; owing primarily to respiration), but there is no suggestion there of spike timing regulation on any faster timescale. Hence, when considered at faster timescales this information is effectively rate-coded (with high OSN convergence ratios onto MOB glomeruli greatly reducing the time required for a reliable estimate of population activity). In contrast, activity in MTCs is structured on the gamma timescale (40–110 Hz). Specifically, mitral cell spikes are phase-constrained with respect to stimulus-evoked gamma rhythmicity (Eeckman and Freeman, 1990; Kashiwadani et al., 1999; Bathellier et al., 2006) that is generated within olfactory bulb circuitry by a sophisticated dynamical system (Li and Cleland, 2017). This metric transformation offers several computational and energetic advantages, and is capable of representing the activation profile across glomeruli using gamma phase in place of aggregate spike rate (Imam and Cleland, 2020). However, any such transformation also provides opportunity to bias the information content of the activated ensemble. This is the essence of selective sampling and the basis for the extraction of useful information from afferent ensembles. However, that said, it is vanishingly unlikely that any single transformation will permit the activity in the follower ensemble to represent one variable of interest exclusively. The physiological tools of transformation are limited, and the environmental variables of potential interest deeply conflated.

For example, current models of olfactory bulb information processing (Cleland and Sethupathy, 2006; Cleland, 2014; Li and Cleland, 2017) require that a measure of concentration tolerance be established within and/or prior to the deep glomerular layer of the olfactory bulb. The dominant effects of stimulus concentration on the breadth and intensity of glomerular activation (Schaefer et al., 2001; Storace and Cohen, 2017) must be reduced for quality-dependent computations to be practically achievable. Indeed, it has long been established that the range of activity in MOB principal neurons is powerfully constrained. Increased odorant concentrations excite some MTCs while inhibiting others (Wellis et al., 1989), and the absolute range of spike rates observed in these neurons is sharply limited with respect to the corresponding ranges of afferent input levels. This degree of concentration tolerance suffices to enable these quality-dependent bulbar computations, and the normalization of activity generates what has been called a *relational* representation among MTCs (Cleland et al., 2007). However, this is different than saying that the MOB circuit achieves concentration *invariance*. Concentration still influences the activity of MOB principal neurons, and hence is also measurable in postbulbar target structures such as the PCx (Bolding and Franks, 2017, 2018). But what is the *effect* of concentration-dependent variance in this projection? Is it (1) a residual contaminant of a quality signal, requiring additional mechanisms within the PCx to achieve a concentration-invariant representation? (2) irrelevant, because afferent inputs to PCx are read out on a different basis (such as spike synchronization; cf. Luna and Schoppa, 2008) that simply bypasses the differences in gross activity attributable to concentration? (3) fed forward to PCx in a physically sophisticated format, such that cortical circuits can easily select for and operate on quality-dominant information while also retaining access to intensive salience, which is an established factor in olfactory learning (Cleland and Narla, 2003; Cleland et al., 2009)? The importance of spike timing regulation on the gamma and beta timescales, which govern both spike integration efficacy and spike timing-dependent learning rules, forces these questions into the forefront. Activity does not necessarily imply meaning, and interpreting neuronal activity patterns based on incorrect hypotheses of coding will lead to qualitatively incorrect interpretations. To understand sensory encoding, activity in any given neuronal ensemble must be analyzed from the perspective of multiple metrics with clear theoretical bases—how much does this activity represent particular sensory variables of interest, and by what activity metrics does it do so? A separate question, then, is to what extent various follower circuits extract and utilize these different bases of information.

### Transforming Variance of Interest

A third essential determinant of sensory processing mechanics is their need for integration with other sensory modalities and brain systems. Both the information architecture that organizes communication among disparate brain systems and the underlying physical mechanisms that directly mediate this communication must be considered when hypothesizing encoding and transformation strategies for particular systems. Two related questions, in particular, should be posed: (1) is there physiological evidence for a common abstract metric space in which multiple modalities or antecedent ensembles can manipulate shared variables? (2) is multimodal integration a discrete, discernable stage of processing, or might it be intercalated deeply into early sensory processing? These questions both relate to the overall question of just how modular and functionally independent we should consider such neural systems to be. How much do local networks insulate one another from a dependence on their many essential local parameters? For example, primary motor cortex in primates appears to encode hand movements as population vectors in veridical space, insulating this common, abstract representational metric from the direct governance of the contractions of many obliquely oriented muscle fibers that control the angles of multiple arm joints and the need to adapt to their non-linear viscoelastic properties (Georgopoulos, 1996; Georgopoulos and Carpenter, 2015). Higher-level olfactory representation often is considered more categorical, hence unlikely to utilize an underlying low-dimensional map akin to these population vectors, but the underlying question remains. How is odor information embedded into useful multimodal representations, and where and how does multimodal information merge with the olfactory sensory cascade?

Odor identification is commonly considered the chief goal of the olfactory system, but it is not clear that this constitutes a singular representational end point. One might instead ask, what are the implications of the sampled olfactory information? A particular odor might have different implications in different contexts, or when paired with specific inputs from other modalities. Should the sensory system be expected to purify the odor identification signal first, and only then coordinate it with other modalities and memories (the classical sensory cortex/association cortex dichotomy)? Or do physiological odor representations depend upon non-olfactory factors at early stages of processing, such that they quickly cease to be strictly “odor representations?”

There is increasing evidence that the latter is the case. In particular, recent work in the anterior olfactory nucleus (AON) has revealed that it is, at least in part, an efferent structure that mediates contextual information from the hippocampus into the olfactory system—very likely into the olfactory bulb itself (Aqrabawi et al., 2016; Aqrabawi and Kim, 2020; Levinson et al., 2020). Direct projections from hippocampal CA1 pyramidal cells also terminate in the MOB granule cell layer (Padmanabhan et al., 2018). That is, sophisticated non-olfactory contextual information is embedded into the olfactory sensory cascade at the same early processing stage that initiates fast oscillatory dynamics (Lagier et al., 2007; Li and Cleland, 2017) and may underlie odor category formation (see *External Plexiform Layer Transformations* section). Moreover, it has already been established that MTC activity reflects olfactory task learning (Doucette and Restrepo, 2008) and the acquired reward value of odors (Doucette et al., 2011; Nunez-Parra et al., 2014), as well as odor quality *per se*. These results suggest a sensory system more aligned toward providing actionable, contextualized information than toward the distillation of invariant odor representations.

The governance of interareal communication in the brain is often based upon transient periods of coherence in higher frequency bands including beta (15–40 Hz) and gamma (Baird and Eeckman, 1993; Fries, 2015). Interactions between the MOB and the PCx appear to be governed by transient periods of oscillatory coherence in the beta band that emerge when MOB gamma oscillations slow down and phase-lock with activity in the anterior PCx (Litaudon et al., 2003, 2008; Cenier et al., 2009; Kay and Beshel, 2010; Frederick et al., 2016). The dynamics of MOB interactions with other structures are less well-characterized, though it has been suggested that the mitral and projecting tufted cells in the MOB interact with two separate populations of granule cells (Orona et al., 1983; Geramita et al., 2016), enabling them to maintain separate and simultaneous LFP oscillations within the MOB at different gamma frequencies (Manabe and Mori, 2013; Frederick et al., 2016). These dynamics in turn could support separate and simultaneous lanes of communication to the PCx and the olfactory tubercle, predicated upon different samplings of the same sensory information transformed into parallel spike timing-based representations. Understanding these physical metrics of information encoding and transfer, along with their governing mechanisms, is critical if we are to understand the selective routing of information across the communication networks of the brain.

## Theoretical Capacities of Olfactory Bulb Circuits

Assessing the capabilities of a complex circuit requires a heavy reliance on theory, and specifically theory that can be challenged and validated across multiple scales and levels of organization. Our early network models examined how OSN populations are able to encode odorants across wide concentration ranges despite the sharply limited dynamic ranges of individual OSNs (Cleland and Linster, 1999), and, once these concentration ranges were captured, how the embedded odor quality information could be segregated from the strongly dominant concentration effects via a sequence of transformations (Cleland et al., 2012), culminating in a more relational pattern of MTC activation (Cleland et al., 2007). In addition to reflecting physiological data (Wellis et al., 1989; Storace and Cohen, 2017), excising the bulk of the mass-action effects of concentration prior to MTC activation is essential for high-dimensional contrast enhancement mechanisms that can distinguish highly similar odorant representations (Cleland and Sethupathy, 2006; Cleland, 2010). A common feature of these modeling approaches is that they focused on Marr's *algorithmic* level of analysis, which defines how an identified computational problem can be solved without necessarily specifying mechanism (Marr and Poggio, 1977; Marr, 1982). The algorithmic function of these transformations (e.g., high-dimensional contrast enhancement, concentration tolerance) must exist in order for the body of theory to stand. However, as discussed below, the particular network mechanisms initially proposed may be partially or wholly wrong without disrupting the larger body of theory.

In contrast, other olfactory systems models are genuinely multiscale, combining algorithmic function with Marr's lowest level of *implementational* detail. These compartmental models require copious constraining data for validation, but are uniquely valuable as existence proofs for the operations of complex physiological systems. For example, fast, synchronous network oscillations in the gamma band have long been recognized as endogenous to the activated olfactory bulb, emerging from the reciprocal synaptic interactions of the external plexiform layer (EPL; Figure 1; Lagier et al., 2007). However, the dynamical system presumed to underlie these oscillations, pyramidal-interneuron network gamma (PING; Traub et al., 1997; Borgers et al., 2005), was not clearly capable of explaining the set of properties exhibited by the olfactory bulb circuit. For example, the local PING frequency is sensitive to the degree of excitatory neuron excitation. Given that substantially different levels of activation among MTCs are the very basis for odor representation, it was unlikely that an unmodified PING mechanism could maintain global synchrony at a common frequency across the entire EPL. Other properties, such as MTCs firing action potentials on some, but not all, gamma oscillations were similarly challenging to square with PING dynamics. Attention eventually turned to the importance of the Type II resonance properties of mitral cells (Desmaisons et al., 1999; Balu et al., 2004; Rubin and Cleland, 2006). Cellular resonance arises from particular balanced configurations of membrane currents, yielding intrinsic subthreshold membrane potential oscillations and imposing a constrained range of favored spiking frequencies. The inclusion of mitral cell resonance properties improved the performance of gamma rhythmicity models in the MOB (Brea et al., 2009), subsequently leading to a modified PRING model for MOB gamma oscillations that highlighted the potential importance of spike timing-based information in the system (Li and Cleland, 2013, 2017; Peace et al., 2018). These are implementational models not simply because of their biophysical detail, but because the questions that they answer are mechanistic. If the membrane and synaptic mechanisms that underlie PRING dynamics prove substantially incorrect, then we no longer understand how broad gamma-band synchronization is achieved in the MOB.

One limitation of both types of purely computational models is that they are only able to study the problems that are appreciated by their creators. Sensory systems deployed “in the wild” have many additional, underappreciated sources of variance to contend with: e.g., interference among odorants from multiple sources (competition for receptors, unknown ligand-receptor efficacies), the adaptation, decay, drift, and/or dropout of receptors, unpredictable fluctuations in odorant concentrations, effects of temperature and humidity, and odor plume dynamics. Testing models with data from physical chemosensor arrays and instantiating biomimetic networks within field-deployable systems provides yet another level of insight into the inconspicuous but essential operations that such systems must perform. Notably, these engineered implementations have revealed a number of problems encountered by our algorithms when deployed “in the wild,” each of which has suggested resolutions that now can be analyzed and experimentally investigated in the biological system. We here summarize and review our common, internally consistent, and broadly data-constrained multiscale framework for sensory processing in the two computational layers of the olfactory bulb, as instantiated in and vetted by computational models and/or engineered neuromorphic systems.

### Glomerular Layer Transformations

The axons of primary OSNs project to the glomerular layer of the MOB, with those of OSNs that express the same odorant receptor converging together to form receptor-specific glomeruli within that layer (Figure 1). The activity of these convergent OSNs is sampled by tens of principal neurons (MTCs) extending apical dendrites into each glomerulus; however, the resulting activation of these MTCs is substantially modified by glomerular-layer computations mediated by a diverse population of interneurons. These computations effect critical signal conditioning functions including contrast enhancement and concentration tolerance, though evidence of structural plasticity among glomerular interneurons further suggests that this layer also mediates additional operations of interest.

#### Concentration Tolerance

The need for substantial concentration tolerance transformations in the early olfactory cascade, prior to MTC activation, has long been clear (Cleland et al., 2007, 2012). As discussed above, however, this does not imply a need for concentration *invariance*. Indeed, MTCs retain a measure of sensitivity to odor concentration changes, and, moreover, mitral and projecting tufted cells differ in the details of their responses to intensity differences (Geramita and Urban, 2017). However, the enormous range of intensity differences encountered under natural circumstances is strongly compressed by early transformations in this system, and these initial models were constructed to elucidate these processes.

A substantial portion of the absolute compression of environmental intensities is probably mediated by a series of mechanisms including axonal convergence, feedback inhibition, and homeostatic synaptic scaling within MOB columns (Cleland et al., 2012). However, it also is essential to supplement these mechanisms with a relational feedback mechanism that normalizes with respect to the total activity sampled *across* columns. This requires a network able to mediate lateral interactions among columns prior to the activation of MTCs, and the only plausible candidate was a lateral network based upon superficial short-axon cells in the deep glomerular layer (Figure 1; Cleland and Sethupathy, 2006; Cleland et al., 2007). Experimental findings at the time indicated that these neurons were excitatory and projected to inhibitory periglomerular cells across multiple MOB columns (Aungst et al., 2003). Based on these data, the proposed mechanism for this relational normalization was a small-world network that leveraged this lateral excitation to build an estimate of mean global activation and then deliver it, via periglomerular cells, onto MTCs as inhibition (Cleland et al., 2007; Cleland, 2010, 2014).

Subsequent experimental work, however, made it clear that short-axon cells are in fact inhibitory interneurons, expressing GABA and dopamine (Banerjee et al., 2015), in this resembling the GAD67-expressing subtype of periglomerular cells (Kiyokage et al., 2010) [Indeed, the multimodal diversity of GABA-expressing interneurons in MOB can be interpreted as a single, distinctly heterogeneous cell group encompassing both the (traditionally defined) periglomerular and short-axon cell types (Sethupathy et al., 2013), though more recent findings indicate a more categorical distinction between presumptive short-axon cells and the remaining diversity of periglomerular cells (Galliano et al., 2018)]. These Banerjee et al. findings entirely superseded the Cleland et al. model predicated on excitatory lateral projections. However, critically, that same work also clearly demonstrated that this network of superficial short-axon cells did, in fact, effect concentration tolerance in the deep glomerular layer as predicted at the algorithmic level of analysis. Moreover, the network elucidated by Banerjee et al. is likely to be more powerful and flexible at its purported task than the small-world model that it replaces. In particular, being based on inhibition, it is more intrinsically stable, and in principle may exhibit a greater capacity to distribute inhibition selectively onto external tufted cells in different MOB columns. That is, in addition to the relational normalization process required for concentration tolerance, there also may be more adaptive, non-uniform transformations, as is suggested by the retention and differentiation of adult-born interneurons in this layer into periglomerular cells (Hack et al., 2005)—though perhaps not short-axon cells (Galliano et al., 2018)—as well as by recent computational modeling work (Zavitz et al., 2020).

#### Non-topographical Contrast Enhancement

Non-topographical contrast enhancement (NTCE; Cleland and Sethupathy, 2006; Cleland, 2010, 2014) was proposed as a mechanism by which highly similar odorant representations could be rendered more dissimilar by surround inhibition, analogous to that observed in retinal circuitry. Notably, genuine surround inhibition had been clearly described in MTC recordings (Yokoi et al., 1995)—as distinct from weaker decorrelation strategies that simply rely upon non-specific activity reductions to reduce overlap among representations. The critical issue of conflict, however, was the metric space in which this surround needed to be mapped. Contemporary assumptions were that the olfactory bulb, like the retina, effected contrast enhancement via nearest-neighbor lateral inhibition based on the physical proximity of MTCs, and that this effect was mediated via inhibitory granule cells. NTCE laid out, on theoretical grounds, why this could not be so. Similarity relationships in odor space are predicated to substantial extent upon similarities in the activation patterns across hundreds of differently tuned receptor types, and therefore exhibit correspondingly high dimensionalities. Systematic changes to these similarity relationships via contrast enhancement consequently need to be determined in that same high-dimensional framework, and certainly could not be adequately approximated by any two-dimensional transformation such as that based on physical proximity in a layered network. However, of course, it was not yet clear how high-dimensional operations could be performed by olfactory bulb circuitry.

**Figure 2:**
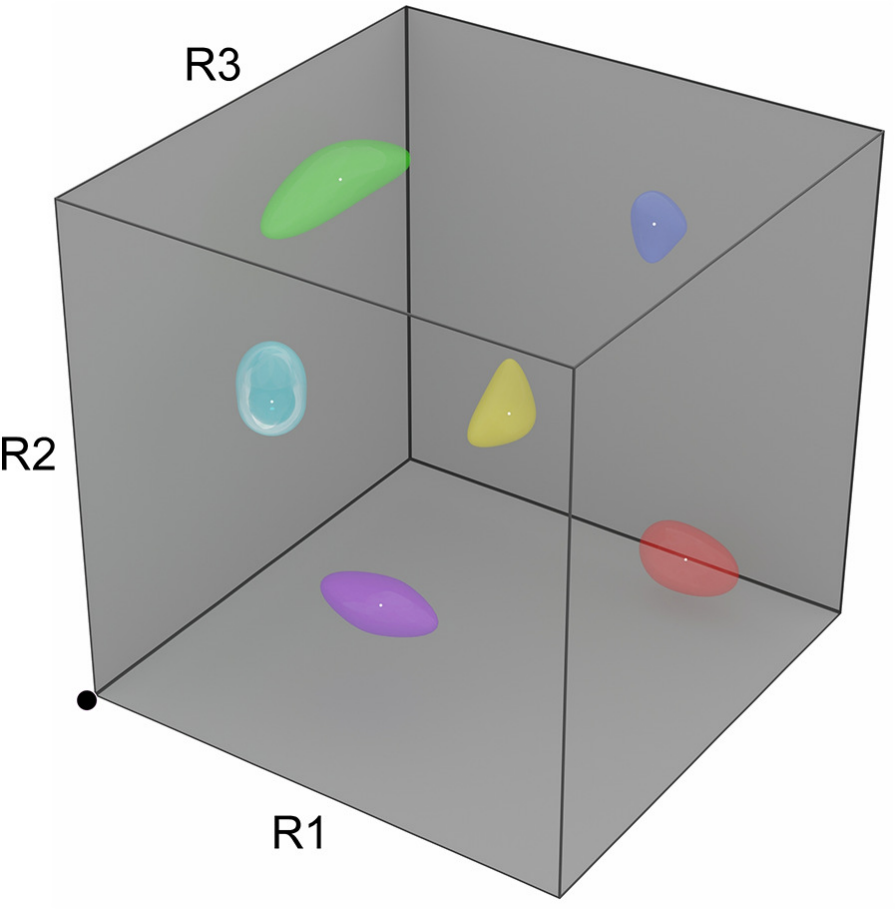
Depiction of a three-dimensional *R*-space. The space of all possible instantaneous odorant representations at the receptor (glomerular input) level can be depicted as a unit *N*-cube, where each axis corresponds to the activation level of one type of odorant receptor, and *N* is the number of odorant receptor types expressed. Note that the numbers of receptor types expressed, as well as their precise structures, are species-specific; accordingly, odorant similarity is an organism-dependent measure. In the three-receptor system depicted, the activation levels of the three receptor types R1, R2, and R3 each independently range from zero at the origin (black dot) to unity (maximally activated). Proximity in this metric space roughly corresponds to odorant similarity (neglecting, for example, learning-dependent effects on perception). Learned odors admit ranges of tolerated variance in the activation levels of each receptor type, reflecting category learning and measurable by generalization gradients; these are depicted in *R*-space as odor source volumes (colored shapes). Importantly, realistic *R*-spaces exhibit much higher dimensionalities than can be depicted here (human *R*-space is ~400-dimensional, and the *R*-spaces of mice and rats are ~1,200-dimensional), such that the distribution of odor source volumes is (1) much sparser than depicted here, and (2) likely to lie along a surface of the unit hypercube, in that odors generally do not activate all of the receptor types in an animal's complement.

The mechanism proposed for NTCE constituted a self-normalization scheme in which the glomerular circuitry of individual MOB columns generated an “inhibitory surround” effect that was directly dependent on overall afferent activation levels. Briefly, when afferent input was weak to moderate, feedforward inhibition would dominate and the MTC would be inhibited, whereas when the input to that column was stronger, excitation would overcome inhibition and drive MTCs to fire. A relational normalization circuit (see *Concentration Tolerance* section) was required in order to render the NTCE mechanism robust to changing odorant concentrations, thereby ensuring that “weak/moderate” and “stronger” inputs in this context would be assessed relative to one another. That is, across an activated ensemble of MOB columns, the MTCs of the most strongly activated columns would fire, those of the more weakly to moderately activated columns would be inhibited below baseline, and the most weakly activated columns would, naturally, generate little physiological response. This algorithm recapitulated the observed phenomenon that, when recording from one mitral cell and moving through its chemoreceptive field by presenting a series of sequentially similar odorants, the MTC would respond with excitation to its preferred odorant stimuli and with below-baseline inhibition specifically to those odorants most similar to its preferred range (i.e., its “surround”). That is, NTCE effected surround inhibition with respect to a high-dimensional odor similarity space that we now term *R*-space (Figure 2; Gronowitz et al., 2020). Moreover, NTCE predicted that this high-dimensional surround inhibition would be mediated by periglomerular interneurons, rather than granule cells, both because only periglomerular cells can effect feedforward intraglomerular inhibition (Figure 1) and because, even with that point aside, granule cells are not well-positioned to suppress MTC spiking activity (Mcintyre and Cleland, 2016). Subsequent experimental and computational work in which the stringency of this contrast enhancement could be regulated by established mechanisms of cholinergic neuromodulation (Mandairon et al., 2006; Chaudhury et al., 2009; Li and Cleland, 2013) lent additional support to this model of olfactory contrast regulation.

Like the concentration tolerance circuit discussed above (see *Concentration Tolerance* section), the NTCE mechanism also requires modification owing to important experimental updates in the structure of glomerular-layer circuitry. However, the essence of its mechanism has been supported by a broad range of subsequent experimental tests. It is now quite clear that mitral cells are gated, and delayed in their activation, by feedforward intraglomerular inhibition delivered by periglomerular cells (Gire and Schoppa, 2009; Geramita and Urban, 2017). Direct tests of the inhibitory control profile of MTCs by feedforward inhibition, including the activation-dependent masking of evoked excitation by evoked inhibition, specifically favored the NTCE mechanism (Fukunaga et al., 2014). The relational normalization process in the deep glomerular layer has been experimentally established (Banerjee et al., 2015). The NTCE prediction that there is no correspondence between MTC chemoreceptive field similarity and physical proximity has been clearly confirmed (Soucy et al., 2009), and the distribution of (functional) lateral inhibitory weights among mitral cells also does not correspond to their proximity (Fantana et al., 2008). However, in contrast to the original NTCE circuit proposal, it has since become clear that the afferent excitation of mitral cells is primarily indirect (Figure 1). Specifically, convergent OSNs activate external tufted cells, which subsequently activate mitral cells within the same glomerulus (Najac et al., 2011; Gire et al., 2012) as well as other glomerular interneurons, coordinating the afferent activation of the entire glomerular microcircuit (Burton, 2017). This is an enormously significant update to our understanding of MOB circuitry with many functional implications. Interestingly, however, it also preserves the algorithmic integrity and even the essential mechanistic principles of NTCE.

Indeed, the indirect mechanism presents advantages to the NTCE framework, alleviating some of its vulnerabilities. For example, NTCE works best when the effects of (disynaptic) feedforward inhibition onto MTCs reliably precede MTC excitation, which had been considered exclusively monosynaptic. This was plausible, in that the exceptionally high input resistance of periglomerular cells and the likelihood that feedforward inhibition was mediated through their gemmules (spines) together facilitated rapid, graded inhibition of MTCs that, if not fast enough to precede MTC excitation *per se*, could be fast enough to precede MTC spike initiation. However, the inhibition computed by the global normalization circuit mediated by short-axon cells would necessarily be slower, requiring the initial inhibitory response to be sustained until this laterally computed feedback inhibition could be incorporated. Mitral cell spike times were in fact sufficiently delayed so as to permit this, and the T-current plateaus observed in periglomerular cells (McQuiston and Katz, 2001) were a potential supporting mechanism for (briefly) sustained inhibition, but this was nevertheless an underspecified weakness in the model mechanism. Indirect MTC excitation via external tufted cells, however, resolves both of these issues, as it slows the process of MTC excitation and additionally fills the role of adjudicating the diverse excitatory and inhibitory inputs to the column, delivering the result of this summation to MTCs. Finally, the indirect framework also simplifies the problem of ensuring that stimulus-activated MTC spikes are robustly constrained by emergent fast oscillatory dynamics. Respiration-synchronized feedforward inhibition evokes a phase reset in mitral cell subthreshold oscillations (Desmaisons et al., 1999; Rubin and Cleland, 2006), which is theoretically important for the reliable recruitment of odorant-activated MTCs into a common gamma regime (Li and Cleland, 2017). Mitral cells that must directly adjudicate inputs from diverse excitatory and inhibitory inputs distributed in time might be less likely to enjoy a common, distinct reset phase across sibling MTCs, or even across the apical dendritic arbor within a single MTC. Whereas MTCs associated with the same glomerulus exhibit heterogeneous properties and non-identical responses, there is evidence that they are coordinated to a certain extent as a common functional unit (Schoppa and Westbrook, 2001).

### External Plexiform Layer Transformations

As the previously purported functions of the EPL now were assigned to the glomerular layer, the question naturally arose: what does the EPL do? It clearly comprises a lateral inhibitory network, but does not respect physical proximity—distributions of functional lateral inhibitory efficacies are largely distance-independent (Fantana et al., 2008). Additionally, accumulating evidence indicated that the EPL exhibited sophisticated synaptic and structural plasticity mechanisms (Balu et al., 2007; Gao and Strowbridge, 2009; Moreno et al., 2009; Lepousez et al., 2013). With appropriate weight distributions, the EPL network theoretically was capable of transforming odor representations in high dimensions, but, as in the glomerular layer, there was no clear intercolumnar similarity metric by which these computations could be framed. Moreover, unlike the glomerular layer, all computations would presumably be based on the specific lateral targeting of inhibition, which would require such an explicit similarity metric in order to, for example, decorrelate representations on the basis of their physical similarities. The exciting possibility then arose that there was no such predetermined metric—that the transformations in this layer were instead governed by plasticity. Under this hypothesis, the EPL would inherit activity patterns that had been transformed by similarity-sensitive glomerular-layer mechanisms, but itself would further transform these representations with respect to distinct, similarity-independent, higher-order bases derived from experience and dependent on network plasticity. Granule cells—which are activated by higher-order combinations of MTC and PCx inputs, but not by inputs from smaller numbers of MTCs (Pressler and Strowbridge, 2017)—could learn these combinations, transiently binding together patterns of receptor activation that together are diagnostic for a given odor, potentially inclusive of its value and/or context. Collectively, then, the EPL would remap the olfactory similarity space inherited from the glomerular layer based on experience and learned utility.

From this, two particular hypotheses stood out. First, the construction of category representations for meaningful “odors” presumably must be initiated in the MOB, where the full spectrum of afferent variance remains computationally available. MOB output diverges into at least three separate pathways, as noted above, and recordings from anterior PCx (for example) suggest that odor representations there already exhibit categorical indicators such as experience-dependent configural responses (Wilson et al., 2006) and discontinuous receptive fields (Stettler and Axel, 2009). Second, receptor occlusion by competing odorant sources is a fundamental, catastrophic challenge to contemporary models of olfactory encoding and representation. Background odorants powerfully disrupt the integrity of olfactory ensemble representations, and are ubiquitous in natural environments. The ability to identify simultaneously-encountered odors of interest in strong and unpredictable backgrounds is essential, and must be resolved early in the sensory cascade. Interestingly, these two hypotheses are likely to interact, as category learning can in principle provide the information needed to identify a known signal presented against a background of strongly disruptive interference.

#### Neuromorphic Design

To instantiate and test these hypotheses, we constructed models of the EPL network and embedded some variants of these models in the experimental Intel Loihi neuromorphic system (Davies et al., 2018). From the engineering perspective, the diagnostic feature of neuromorphic networks is a reliance on local computation; no global error term is assessed, and the weight of a synapse is modifiable only by the activity experienced directly by that synapse. Neuromorphic algorithms also tend to rely on spike-based communication (i.e., they operate as pulsed networks; Maass and Bishop, 1998), though this is not necessarily diagnostic. Finally, they often rely upon task-specific network architectures, and hence can be much faster to train at the cost of generality (Imam and Cleland, 2020). This combination of features enables neuromorphic hardware platforms to execute appropriate neuromorphic algorithms rapidly and with very low energy expenditure, notably by colocalizing memory and compute resources on-chip to leverage the benefits of local computation. A still-underappreciated advantage of neuromorphic design is the ease with which the properties of nodes (cells) and edges (synapses) can be arbitrarily heterogeneous and elaborated, and networks can fork, merge, and loop datastreams. These features, however, are difficult to optimize, and hence place substantial burdens upon the designer. Biologically inspired network motifs fill this gap admirably, as the biological systems—to the extent that they are correctly understood—provide existence proofs for the functionality and robustness of the network architecture. Conversely, and with the same caveats, physically instantiated biomimetic architectures tested with real-world datasets can challenge our understandings of biological systems and present quantitatively vetted hypotheses for the next iteration of conceptual frameworks.

#### EPL Network Plasticity Enables Rapid Learning and Odor Identification Under Noise

We constructed a network model of the MOB external plexiform layer based on its theoretical capacities and key circuit motifs in order to address the problems of (1) how categorical odor representations are generated and (2) how these might then be leveraged to identify known odor sources within highly interfering and unpredictable backgrounds (Imam and Cleland, 2020). Briefly, if the plastic synaptic matrix of the olfactory bulb EPL (Figure 1) can be leveraged to construct categorical odor representations, sparsely distributed within a high-dimensional receptor-based similarity space (*R*-space; Figure 2; Gronowitz et al., 2020), then an attractor network (Hopfield, 1982; Kay et al., 1996) should be capable of identifying a known odor despite this disruptive interference. Notably, the high dimensionality of this olfactory *R*-space is of great benefit, both because the greater number of receptor types increases the likelihood that some critical fraction will remain free of catastrophic occlusion, and because higher degrees of freedom means that random disruptive interference is increasingly unlikely to resemble a different learned odor representation.

To construct this network model, we instantiated some core principles of MOB computation that we have hypothesized for the biological system (Cleland, 2014; Li and Cleland, 2017). These include:

1. The importance of gamma-discretized spike timing-based computation in the EPL. This enables the direct deployment of asymmetric spike timing-dependent plasticity (STDP) rules, which in turn enable the construction of higher-order, configural receptive fields in granule cells (Linster and Cleland, 2010). PCx pyramidal neurons also are known to be selective for synchronized MTC spikes on this timescale (Luna and Schoppa, 2008).
2. The principle that that only a minority of principal neurons participate in gamma dynamics during any given stimulus presentation, based on their afferent activation (Li and Cleland, 2017). MTCs are known for their relatively high background spiking activity (a side effect of their high sensitivity to inputs), but this background activity does not correspond to MTCs' responsivity to odor stimuli (Kollo et al., 2014), nor do background spikes align to the common, coherent gamma rhythm (Werth and Cleland, 2019). The synchrony-selective mechanisms of point (1) suggest that MTC activity that is disorganized in time simply fails to effectively activate follower neurons.
3. The principle that GC inhibition of MTCs manifests as *delays* in MTC spike times on the gamma timescale (inhibition-imposed phase lag). This counteracts the phase lead exhibited by more strongly activated MTCs owing to their more rapid escape from inhibition on each gamma cycle, thereby recapitulating the direct opposition of cellular excitation and inhibition within a gamma-scale spike timing regime. This metric transformation neatly avoids the catastrophic limitation of rate coding assumptions in sparsely active networks like the MOB: the need for postsynaptic neurons to integrate over extended periods of time in order to accurately estimate presynaptic activity.
4. The principle that the topology of the EPL network is not reciprocal, despite observations that individual synaptic connections between GCs and MTC lateral dendrites often are bidirectional. Specifically, MTC lateral dendrites project broadly across the EPL (Mori et al., 1983; Orona et al., 1984) and support regenerative spiking, and hence can deliver excitation to GCs independent of proximity (Xiong and Chen, 2002). In contrast, inhibition does not propagate; for GCs to affect spike timing propagation in MTCs, they must deliver their shunting inhibition very close to the afferent axis (i.e., the MTC soma; Mcintyre and Cleland, 2016). This facilitates the abstraction of MOB *columns*, each consisting of a glomerulus, its associated principal neurons, and the GCs that deliver effective inhibition onto those principal neurons. Transsynaptic tracing studies suggest just such a discontinuous and column-centric topology of MTC-GC connectivity (Willhite et al., 2006; Kim et al., 2011).
5. The principle that, within the constraints of this asymmetry, the topology of the lateral inhibitory network of the EPL is dynamically acquired and learning-dependent. Specifically, GCs learn higher-order configural receptive fields, becoming more specifically diagnostic of particular odorants than is possible for principal neurons, and then deploy this information to regulate MTC spike timing. Consistent with this principle, GCs require either coincident activity by a large number of MTCs, or by a smaller number of MTCs coupled with excitation from PCx, in order to fire reliably (Pressler and Strowbridge, 2017). Moreover, task learning is known to shape MTC activation profiles (Doucette and Restrepo, 2008), and acquired odor-reward associations regulate MTC spike synchronization patterns (Doucette et al., 2011).
6. The corollary to point (5) that GCs are permanently differentiated by recruitment during the process of odor learning, and therefore require constitutive replacement by adult neurogenesis in order to enable future (lifelong) learning. Such a strategy for category learning may explain why GCs outnumber MTCs by roughly 25:1 (Meisami and Safari, 1981; Frazier and Brunjes, 1988; Royet et al., 1998), whereas most brain regions exhibit comparable numbers of excitatory and inhibitory neurons, or even a bias toward the excitatory (Shepherd, 2004).
7. The possibility that neuromodulation should be conceived as an optimization trajectory rather than as a stationary, behaviorally-dependent state.

This combination of elemental structures and properties enabled the construction of a neuromorphic network that, for the first time, successfully addressed the problem of identifying odor sources despite powerful interference by simultaneously encountered background odorants within a complex chemical environment (Imam and Cleland, 2020). Odor presentations were drawn from the responses of a 72-chemosensor array to stimulation with a complex odorant plume in an wind tunnel (Vergara et al., 2013). Background interference included both Bernoulli and quasi-Gaussian components. Specifically, Bernoulli noise was delivered by replacing the activity levels of some fraction of the sensors (typically 40–60%) with random numbers. This process models competition for receptors by other, unknown odorant species that may strongly activate, weakly occupy, or block the activation of any given receptor in an unpredictable manner (Araneda et al., 2000; Gronowitz et al., 2020). Quasi-Gaussian noise was drawn directly from the plume dynamics of the odorants as encountered, and imposed additional variance on the output of all receptors. Networks trained on all 10 different odorants with one-shot learning reliably identified all 10 noise-occluded test odors. Briefly, local spike timing-dependent learning rules at excitatory and inhibitory synapses generated fixed-point attractors that, when presented with occluded test odorant signals, iteratively modified the activity pattern via recurrent inhibition over multiple gamma cycles until the known odor could be clearly identified (Imam and Cleland, 2020).

Training using this algorithm is rapid—even one-shot learning suffices to learn a new odorant signature. Moreover, the network naturally exhibits *online learning*—the capacity to learn multiple inputs (odors) sequentially, without the new training causing the catastrophic forgetting of previous training. Both of these properties stand in sharp contrast to the properties of contemporary deep networks. Specifically, a similarly sized deep network required thousands of training trials, plus foreknowledge of the variance structure of the interference, in order to reach the same levels of task performance as this neuromorphic strategy, and also lacked the capacity for online learning (Imam and Cleland, 2020). These properties of the EPL network model parallel those of the biological system: rapid learning, robust identification under noise, and the potential for sustained performance across unpredictable or changing environments.

This implementation demonstrates that the circuitry of the mammalian MOB is, in principle, capable of supporting odor category learning (potentially inclusive of context and valence, as discussed above) and of using these categorical memories to facilitate odor recognition in complex milieus. The particular benefit is that category learning here is not simply a cognitive outcome, but also can be applied very early in the sensory cascade in order to achieve an extraordinarily difficult sensory goal. That said, there are critical simplifications in this initial implementation that must be superseded with further development. Behavioral studies of learned odor generalization (Cleland et al., 2009, 2012) have indicated that incipient odor categories adapt to include ranges of quality variance that have similar implications—that is, odor sources of interest (like “orange”) include ranges of variation in quality that can be genuinely random or be diagnostic of within-category properties such as ripeness or cultivar. Odor representations, then, constitute smooth manifolds in *R*-space (Figure 2) rather than fixed points, and their categorical boundaries are probabilistic rather than clearly discrete. The fixed point attractors used in the initial model consequently need to be replaced with some form of manifold learning, inclusive of intracategorical hierarchy. Rather than one-shot learning, these manifold learning strategies will respond to increased training experience with correspondingly more reliable statistical estimates of category variance (Cleland et al., 2009; Cleland, 2014). Similarly, the current form of the inhibitory learning rule forestalls manifold learning; its more sophisticated replacement may require expansion of the network to include the feedback loop between MOB and PCx, which in the biological system supersedes intrinsic MOB gamma with slightly slower (beta band) coherent oscillations between these two structures (Cenier et al., 2009; Frederick et al., 2016). Indeed, existing efforts to study the neuroscience of odor categorization have focused on the PCx and its associated limbic regions (Bao et al., 2016; Qu et al., 2016), and analogous studies in insects also suggest that categorical processing incorporates plasticity in peripheral networks together with higher-order olfactory structures (Locatelli et al., 2016; Strube-Bloss and Rossler, 2018). Finally, the rules by which adult-generated interneurons are incorporated into the neuromorphic network also are simplified, privileging strict categorization over similarity-dependent generalization processes. Nevertheless, the successes of this prototype provide strong hypotheses for the operations of the biological olfactory system that other experimental and theoretical studies have not to date suggested.

#### Learning in the Wild

The initial EPL model proved robust to the amount of variance associated with the odor plume together with a measure of background occlusion. However, as discussed above, biological systems are subject to many additional sources of uncontrolled variance. Concentrations vary widely, sensors decay, and the network must continually update representations according to newly acquired information. Moreover, the goals of odor learning include not simply classification into one of a set of discrete categories, but the recognition of similarities among diverse stimuli sufficient to form generalization gradients (Shepard, 1987; Cleland et al., 2002, 2009) and generate hierarchical category representations (Edelman and Shahbazi, 2012; Clapper, 2019). Modeling these capacities in artificial systems robustly screens alternative hypotheses for efficacy in the real world. The overarching capacity to learn multiple odors rapidly and sequentially, recognize diverse odors across concentrations and under highly occluding circumstances, adapt to the breakdown of sensors, apply perceptual learning processes to update representations and manage similarity, and remain adapted to an unpredictable or evolving environment is summarized as *learning in the wild*.

Some aspects of learning in the wild simply require the inclusion of established glomerular-layer computations affording concentration tolerance and regulated contrast enhancement (Imam et al., 2012; Borthakur and Cleland, 2019a,b). Enabling the explicit representation of similarity further requires that we loosen the strict controls over the allocation of adult-born neurons used in the Loihi model (Imam and Cleland, 2020), thereby enabling granule cell interneurons to belong to multiple ensembles according to the similarity profiles among learned odor representations. Early instantiations of this richer network have been promising, but also have presented challenges highlighting two additional principles that improve network robustness and performance: parameter heterogeneity and data regularization. Briefly, fully instantiated networks can be sensitive, performing best when presented with well-behaved inputs that statistically resemble those against which they were initially parameterized. Signal conditioning mechanisms that afford concentration tolerance are critical in this regard (Borthakur and Cleland, 2019b), and contrast enhancement (whether static or deployed in a trajectory) can improve the statistical match between a sample and the network (Imam and Cleland, 2020). In addition to these transformations, heterogeneity in specific model parameters can improve the robustness of odor identification and increase the diversity of inputs that the network can effectively process. Heterogeneity in GC spike thresholds, for example, generates a controllable range in the order of their post-differentiation receptive fields, rendering some highly selective for specific known odorants and others more broadly tuned. Similarly, the duplication of MTCs in each MOB column, coupled with heterogeneity in their excitability properties, facilitates the statistical regularization of sensory input data, thereby enabling the network to respond effectively to a wider diversity of sensory inputs (Borthakur and Cleland, 2019a). In neuromorphic systems, these properties enable single, parameterized networks to function effectively when presented with widely disparate datasets—from chemosensor arrays with years of accumulated drift and decay (Borthakur and Cleland, 2019b) to non-olfactory datasets with diverse input statistics (Borthakur and Cleland, 2019a).

Heterogeneity in cellular and synaptic properties is of course expected in biological systems, but there is increasing evidence that this is a cultivated property of neuronal networks rather than arising solely from inescapable random variability. Indeed, heterogeneity in cellular and synaptic properties has been credited with improving the sensitivity, efficiency, and information content of neuronal ensembles (Lengler et al., 2013; Zohar et al., 2013) and regulating the balance between network robustness and flexibility (Gu et al., 2019). The advantages of parameter heterogeneity observed in artificial systems models are indicating a broader emerging principle of biomimetic design: considerable computational power and robustness may be achievable by relaxing control over critical variables and subsequently reconstructing the required specificity through effective sampling strategies.

## Conclusions

Understanding any information processing system requires insight into the problems that it faces, and the forms that possible solutions to these problems can take (Marr and Poggio, 1977). Hence, the role of theoretical neuroscience is not only to replicate and integrate established results, but to extend beyond contemporary experimental findings so as to inspire and guide subsequent investigations. Engineered systems models expand our capacity to identify complex theoretical problems and limitations that neither neurobiological experiments nor more narrowly targeted models are able to appreciate. Such models can be speculative, but are at their most powerful when they analytically cross-reference information from multiple sources, constraining the possible interpretations more sharply than any of the individual contributing studies are able to do. This is doubly true when such models incorporate complex, multi-stage systems within a single, internally consistent, quantitative framework. Some problems are only addressed by sequences of mechanisms coordinated across stages. Some other problems are best solved by relaxing the diagnostic effort at one stage so as to improve the capacity of a subsequent stage to achieve that diagnostic goal in a flexible and task-dependent manner. Biological networks exemplify this multistage, systems approach to sensory sampling and information acquisition. A corollary of this position is that narrower, stage-specific analyses are at risk of misinterpreting the computational and “coding” strategies of the system.

We propose a common, integrated theory of olfactory systems operation that incorporates experimental findings across scales, stages, and methods of analysis into a common framework. This constructive approach to systems analysis considers individual stages of processing in a broadened context as contributors to global goals, while still generating concrete, stage-specific hypotheses that can be falsified or updated by experimental results. By leveraging the test platforms and analytical capabilities of engineered approaches together with our expanding capacity to interrogate biological systems, contemporary theorists can design, implement, and validate increasingly sophisticated and integrated theoretical models of neural and cognitive systems.

## Data Availability Statement

The original contributions presented in the study are included in the article/supplementary material; further inquiries can be directed to the corresponding author.

## Author Contributions

TC developed the general theory and wrote the paper. AB developed and analyzed olfactory bulb models and contributed to the writing of the paper. All authors contributed to the article and approved the submitted version.

## Conflict of Interest

The neuromorphic algorithm described in the *EPL Network Plasticity Enables Rapid Learning and Odor Identification Under Noise* section is the subject of a Cornell University patent application on which both authors are listed as inventors. TC is a member of the Intel Neuromorphic Research Community and has received research funding from Intel for related work. The authors declare that this study received funding from Intel. The funder was not involved in the study design, collection, analysis, interpretation of data, the writing of this article or the decision to submit it for publication.
